# Fluoride Varnish as Root Canal Sealer: A Scanning Electron Microscopy and Bacterial Penetration Study

**Published:** 2014-12-24

**Authors:** Masoud Parirokh, Mohammad Talebizad, Farshid Reza Forghani, Ali Akabar Haghdoost, Saeed Asgary, Mohammad Jafar Eghbal, Jamileh Goddousi

**Affiliations:** a * Kerman Neuroscience Research Center, Institute of Neuropharmacology, Kerman University of Medical Sciences, Kerman, Iran; *; b * Private Practice, Tehran, Iran *; c * Oral and Dental Diseases Research Center, Kerman University of Medical Sciences, Kerman, Iran; *; d * The Research Center for Modeling in Health, Institute for Future Studies in Health, Kerman University of Medical Sciences, Kerman, Iran; *; e * Iranian Center for Endodontic Research, Research Institute of Dental Sciences, Shahid Beheshti University of Medical Sciences; *; f * Department of Endodontic, Dental School, Mashhad University of Medical Sciences, Mashhad, Iran*

**Keywords:** Bacterial Leakage, Fluoride Varnish, Root Canal Sealer, Scanning Electron Microscopy, SEM

## Abstract

**Introduction:** This study was carried out to evaluate the bacterial leakage of root canal fillings when cavity varnish containing 5% fluoride (Duraflur) was used as root canal sealer. **Methods and Materials:** Root canals of 88 straight single-rooted teeth were prepared. Eighty teeth were randomly divided into 3 experimental groups (*n*=20) and two positive and negative control groups of ten each. The roots in group I and II were obturated with gutta-percha and AH-26 sealer using lateral condensation technique. The root canal walls in group II were coated with a layer of varnish before obturation. In group III the canals were obturated with gutta-percha and fluoride varnish as the sealer. *Enterococcus faecalis* (*E. faecalis*) was used to determine the bacterial leakage during 90 days. The Kaplan Meier survival analysis was used for assessing the leakage and log rank test was used for pairwise comparison. The rest of eight single rooted teeth were selected for scanning electron microscopy (SEM) evaluation with 5000× magnification. **Results: **Leakage occurred between 20 to 89 days. Group III showed significantly less bacterial penetration than groups I and II (*P*=0.001 and *P*=0.011, respectively). However, there was no significant difference between group I and II (*P*>0.05). SEM evaluation showed that the varnish had covered all dentinal tubules. **Conclusion: **The present study showed promising results for the use of fluoride varnish as root canal sealer but further *in vitro* and *in vivo* studies are needed.

## Introduction

The purpose of root canal obturation is to prevent leakage of bacteria and their byproducts into the root canal system and finally to the periradicular area. Complete obturation of the pulp space is an important part of root canal therapy. The ideal material used for this purpose should be inert, dimensionally stable, biocompatible, non-toxic and radiopaque. Moreover it preferably should have known solvents [1].

Numerous investigations on a large number of filling materials have been reported [2-8]. Gutta-percha is the most widely used material for root canal filling. However, it does not adhere to the root canal walls and provides poor sealing, so it should be used in combination with a root canal sealer to improve sealing and obturation quality [1]. A large number of root canal sealers have been tested in combination with gutta-percha in an attempt to provide a tight seal [3, 9-12].

Use of fluoride varnishes has been recommended as a prophylactic solution against dental caries [13], surface treatment of delayed replanted avulsed teeth [14], treatment of dentin hypersensitivity [15-17] and as a root canal filling material for primary teeth in combination with zinc oxide and calcium hydroxide (CH) [18].

In an *in vitro* study, Mickel *et al.* [19], showed that stannous fluoride inhibited the growth of *Enterococcus faecalis (E. faecalis*) significantly more than CH and a combination of both. They suggested that stannous fluoride might be a good material for dressing root canals between appointments [19]. Sen and Buyukyilmaz [20] evaluated the effect of titanium tetrafluoride solution on root canal walls using scanning electron microscopy (SEM). SEM images revealed that fluoride solution occlude the dentinal tubules. They concluded that using titanium tetrafluoride solution might prevent recontamination of dentinal tubules [20]. Duraflur (Pharmascience, Montreal, Québec, Canada) is a sodium fluoride varnish containing 5% fluoride, that was also successfully used for preventing caries and reducing teeth hypersensitivity [21].

The purpose of this *in vitro* study was to evaluate the sealing of root canal obturation after application of Duraflur varnish as root canal sealer, using microbial leakage test and SEM observation.

## Methods and Materials

In the present study 88 freshly extracted single rooted teeth that were extracted either because of periodontal problems or for orthodontic reasons were used. The teeth were resected at the CEJ level with a high-speed diamond bur under water irrigation. The roots were inspected for crack and resorption under a stereomicroscope (Wild M5A, Heerbrugg, Switzerland). For root canal sterilization all roots were autoclaved. The methods of the assessments were as follows.

***Bacterial penetration***

The root canals of the 80 teeth were cleaned and shaped using the crown-down technique with K3 instruments (SybronEndo, Orange, CA, USA). The working length was visually determined 1 mm shorter from the anatomical apical foramen. The root canals were irrigated with 3 mL of 5.25% sodium hypochlorite (NaOCl) within instrumentation. Prior to obturation the smear layer was removed by intermittent irrigation with 5 mL 17% EDTA (Asia Chemi Teb. Co., Tehran, Iran) for 1 min and 5 mL 5.25% NaOCl. The roots were then randomly divided into three experimental groups (*n*=20) and negative and positive control groups of 10.

The roots in group I were filled with gutta-percha and AH-26 sealer (Dentsply, De Trey, Konstanz, Germany). The root canal walls in group II were coated with Duraflur varnish (Pharmascience, Montreal, Québec, Canada) and then were obturated with gutta-percha and AH-26 sealer. The roots in group III were coated by Duraflur varnish as root canal sealer that was carried into the canal using lentulo spiral (Dentsply Maillefer, Ballaigues, Switzerland). Then they were obturated with gutta-percha. For all samples, lateral condensation technique was the method of obturation.

All samples in the negative control group (10 roots) were covered completely with two layers of nail polish including the apical portion. Positive control samples consisted of 10 unfilled roots. The external surfaces of the specimens were covered with two layers of nail polish from the coronal edge to 1 mm short of the anatomic apex. The roots were inserted individually into Eppendorf tubes with the root apex protruding through the cut end of the tube. The coronal and middle portion of each specimen was sealed with cyanoacrylate glue to prevent leakage at the connection zone. Care was taken to ensure that no cyanoacrylate glue covered the coronal end of the root. The specimen were sterilized using ethylene oxide gas and placed in a cryo sterile bottle containing 3 mL sterile BHI (brain-heart infusion) broth (Merck, Darmstadt, Germany) with phenol red to ensure that the apical portion of root was located in liquid. The coronal chamber was inoculated with 0.5 mL of BHI containing approximately 10^ 6^ *E. faecalis* (ATCC 8213) per 1 mL, using a sterile syringe and 22-gauge needle. The medium with microorganisms was changed every three days. The system was stored in an incubator at 37^°^C and color change of the culture (red to yellow) in the apical chamber was checked every day for three months. The time taken for this to occur was recorded as an indicator of coronal contamination. Cultures from the apical chamber were streaked onto BHI culture plates. Microorganisms were identified by colony morphology gram stain and catalase test.

The Kaplan Meier survival analysis was used for calculating the median time of leakage and the log rank test was for pairwise comparison of groups. The level of significance was set at 0.05.

***Scanning electron microscopy***

Eight roots were used for SEM examination. After root canal preparation and irrigation in a similar manner to the bacterial leakage study, the canals were dried with paper points and the roots were longitudinally bisected with pliers after making longitudinal grooves on the labial and lingual root surfaces with a diamond disk. After splitting the roots, in one half a layer of Duraflur varnish was applied on to the root canal wall while the other half was used as control. The varnish samples were left at room temperature for one week under coverage. Two samples of the varnish group were randomly selected and splitted into two halves to evaluate varnish penetration inside the dentinal tubules. Then all samples were put in an oven and were dried at 50^°^C for 24 h. 

**Table 1:** Frequency and mean (SD) of bacterial penetration in different groups

**Group (N)**	**Contaminated N (%)**	**Uncontaminated N (%)**	**Mean (SD) **
**AH-26 (20)**	11 (55)	9 (45)	79.1±4.37
**AH-26+Duraflur (20)**	8 (40)	12 (60)	80.2±4.50
**Duraflur (20)**	1 (5)	19 (95)	86.65±3.27
**Control+ (10)**	10 (100)	0 (0)	-
**Control-(10)**	0 (0)	10(100)	-

**Figure 1:**
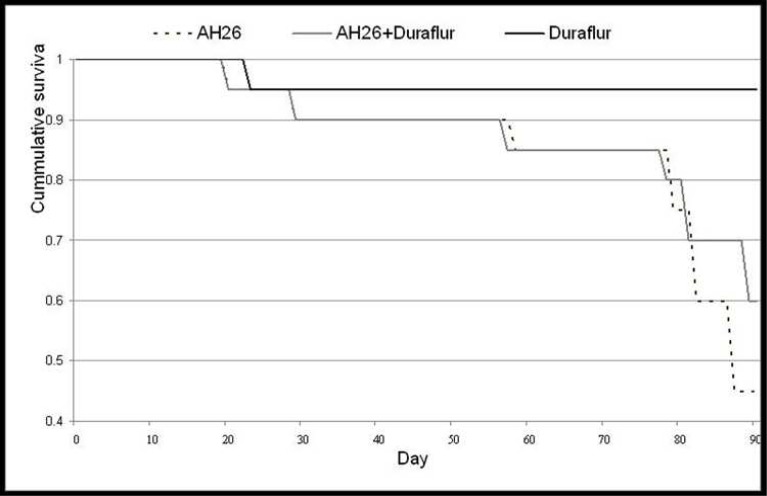
The plot shows bacterial penetration in different groups

After drying, all samples were gold-coated using a K550X sputter coater (Emitech Ltd, Ashford, UK). An electron microscope (Stereoscan 360, Cambridge Instruments, Cambridge, UK) was used for imaging and observing the basic morphology of dentinal tubules at an accelerating voltage of 15-20 kV. The specimens were observed with 5000× magnification.

## Results

***Bacterial leakage***

All samples in the positive control group exhibited bacterial leakage within 24 to 48 h, while the lower chamber of all negative control teeth remained uncontaminated throughout the study. The highest amount of leakage was found in group I (11 samples, 55%) followed by group II (8 samples, 40%) and group III (1 sample, 5%). Only one sample in group III showed leakage at 23^rd^ day. The first appearance of bacterial leakage was observed at 20 days for groups I and II. The average time for bacterial penetration among groups in descending order was: AH-26>AH-26+Duraflur>Duraflur (Table 1).

The Kaplan Meier survival test (Figure 1) showed that there were significant differences between bacterial penetration in group III in comparison with group I (*P*=0.001) and group II (*P*=0.011). There was no significant difference between groups I and II (*P*=0.406).

***SEM examination***

**Figure 2:**
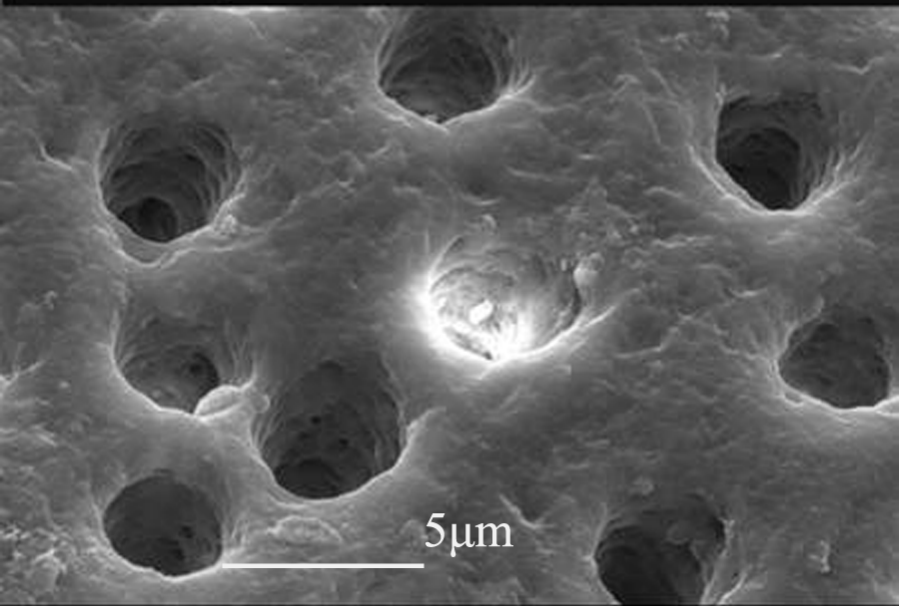
Patent dentinal tubules in control group (5000× SEM magnification)

**Figure 3:**
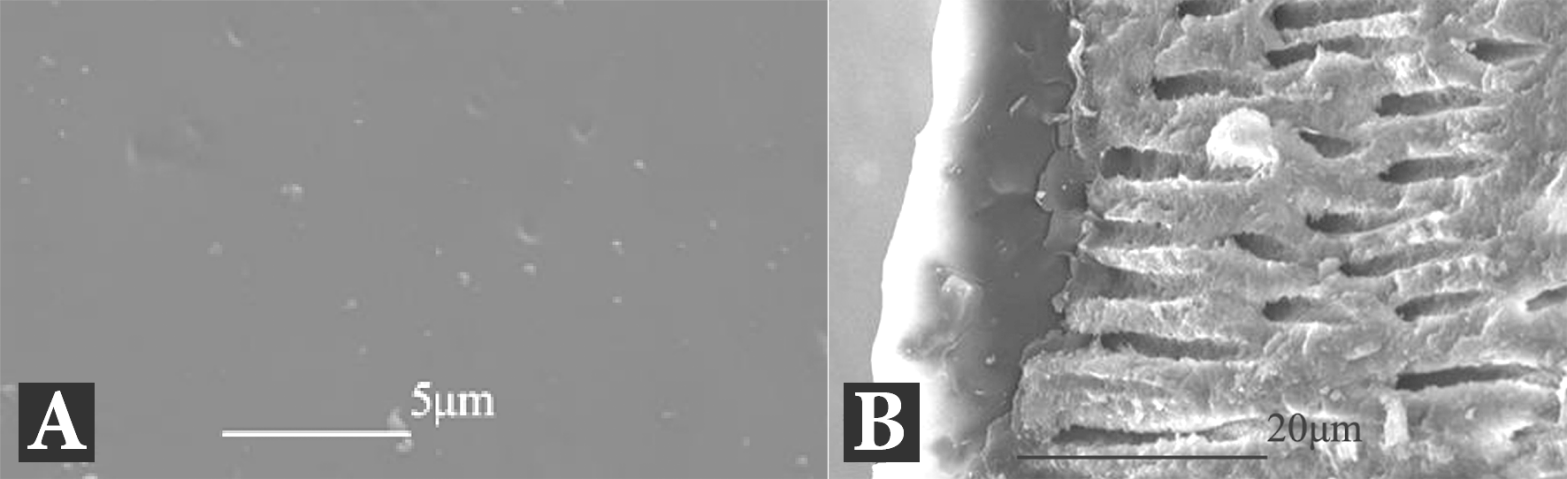
*A)* The root canal wall covered with varnish (5000× magnification); *B) *Varnish penetration inside the dentinal tubules (2500× magnification)

All control specimens showed clean patent dentinal tubules (Figure 2) whereas samples that were covered with Duraflur varnish showed a smooth surface within which all dentinal tubules in all parts of the root canals were covered (Figure 3A). Split specimens showed a 10-15 μm-thick varnish layer covering the surface of dentinal tubules. Also limited penetration of Duraflur varnish inside the dentinal tubules could be observed (Figure 3B).

## Discussion

The results of the current study showed that when fluoride varnish (Duraflur) was used as root canal sealer, more resistance to bacterial penetration could be expected (*P*<0.05). SEM observation showed total coverage of dentinal tubules by varnish at all parts of the root canal.

Tight seal in either apical or coronal region of the root canal is important for gaining clinical success [1]. Therefore, leakage studies on various root canal obturation materials and techniques, root-end filling and furcation repair materials provide the basis for contemporary endodontic research. A number of leakage models have been described in the literature, including penetration of tracers such as dye, radioisotopes, microorganisms and their toxins, and fluid filtration method [22-26]. In this study bacterial penetration test was used because microorganism leakage tests are commonly used for evaluating the sealing ability of root canal sealers and appear clinically relevant [4, 27, 28].

A meta-analysis on *in vitro* published studies by Shahravan *et al.* [29], concluded that smear layer removal improved fluid tight seal of the root canal obturation. Therefore, in this study smear layer was removed prior to root canal obturation.

In the present study *E. faecalis* was selected as the test bacteria because it is a part of normal human microflora and is frequently cultivated from the root canals with failed endodontic treatment [27, 30, 31]. In addition, many research studies have used *E. faecalis* as the test bacteria [2, 9, 10, 32]. Many bacterial penetration studies have shown that the average numbers of days for total bacterial penetration might be similar for different groups during the experiment, while there might be a very wide range of bacterial penetration for all groups [8, 9, 33, 34]. In this study, the same results were obtained (average of bacterial penetration was 79.1 days for group I, 80.2 days for group II, and 86.65 days for group III). Saunders *et al. *[34] believe that bacterial penetration can be attributed to variable root canal anatomy, method of canal preparation and sealer type. In this study, due to using similar method of preparation and comparable root canal anatomy the differences in bacterial penetration among the groups can be attributed to the difference of root canal sealing method.

Sen and Buyukyilmaz [20] in a SEM study have used 4% titanium fluoride solution for covering the root canal walls in presence and absence of smear layer. Results of their study showed that the solution could successfully seal off the dentinal tubules. In the present study, despite using of a different material (5% fluoride varnish) the similar finding was obtained. SEM images showed that no patent dentinal tubule could be observed after varnish application.

The U.S. Food and Drug administration (FDA) has cleared fluoride varnish for the treatment of dentin hypersensitivity associated with the exposure of root surfaces or as a cavity varnish. This can justify the idea of intracanal tubular sealing by varnishes, as well. Meanwhile, the American Dental Association (ADA) has endorsed the use of fluoride varnish for caries prevention, however, its application as a root canal sealer has not been cleared by FDA, yet [35].

## Conclusion

Fluoride varnish showed reasonable root canal sealing ability compared to AH-26 root canal sealer. Therefore, it can be assumed that fluoride varnish has the potential to be a root canal sealer. Further investigations are needed to evaluate the material’s biocompatibility and physical properties.
